# Impact of Guided Endodontics on the Success of Endodontic Treatment: An Umbrella Review of Systematic Reviews and Meta-Analyses

**DOI:** 10.7759/cureus.68853

**Published:** 2024-09-07

**Authors:** Aakansha Puri, Dax Abraham, Alpa Gupta

**Affiliations:** 1 Department of Conservative Dentistry and Endodontics, Manav Rachna Dental College, Manav Rachna International Institute of Research and Studies (MRIIRS), Faridabad, IND

**Keywords:** cone beam computed tomography, endodontics, guided endodontics, microsurgery, navigation system

## Abstract

Endodontic treatment, essential for preserving teeth affected by pulp or periapical diseases, often encounters challenges such as complex root canal anatomies and calcifications that can hinder success. Traditional techniques, although effective, are limited in addressing these complexities. Guided endodontics, which utilizes advanced imaging and navigation technologies, promises enhanced accuracy and precision, potentially improving treatment outcomes. Thus, this umbrella review aims to assess whether guided endodontics influences the outcome of endodontic treatment by synthesizing evidence from multiple systematic reviews and meta-analyses. Comprehensive searches were conducted in PubMed, Scopus, EBSCOhost, and Cochrane databases to identify relevant studies. Additionally, grey literature was searched. The umbrella review protocol was developed and registered in the International Prospective Register of Systematic Reviews (PROSPERO) (CRD42024564150). Inclusion criteria comprised randomized controlled trials (RCTs), randomized clinical trials, case series, and case reports. Exclusion criteria included reviews, non-English language articles, in vitro studies, ex vivo studies, randomized experimental (in vitro) studies, narrative reviews, and expert opinions. Studies involving patients undergoing guided endodontic treatment with dynamic and static navigation systems were included. The interventions aimed at improving the accuracy and precision of endodontic procedures, particularly in challenging cases such as locating calcified canals and performing microsurgery. The study involved three reviewers who independently appraised the systematic reviews for eligibility, data extraction, and review quality. The quality of the systematic reviews was evaluated using the “A Measurement Tool to Assess Systematic Reviews 2” (AMSTAR2) assessment tool. The methodological quality of the systematic reviews was assessed. A pooled review of the studies showed that guided endodontics demonstrated more precise and better results than conventional endodontic procedures.

## Introduction and background

Endodontic therapy is an essential treatment modality to preserve natural teeth affected by pulp pathology. Despite its success, challenges still prevail, such as pulp calcifications, periapical lesions, iatrogenic perforations, fractures, excessive tissue removal, inability to remove instrument fragments, and difficulty navigating heavily calcified canals. Although magnifying glasses, microscopes, and cone-beam computed tomography (CBCT) can provide guidance, it can be challenging for operators to perform the therapy manually without errors [1].

Guided endodontics (static navigation (SN) and dynamic navigation (DN)) uses computer-aided navigation systems to aid in accurately locating and treating root canals. These technologies produce a visual guide that helps with precise endodontic procedures by combining real-time navigation with preoperative imaging, such as CBCT, thereby increasing the accuracy and predictability of endodontic procedures [1,2]. Given the conflicting results of existing systematic reviews and meta-analyses on the effect of guided endodontics on endodontic procedures, the aim of this umbrella review is to evaluate the potential impact of guided endodontics on the outcome of endodontic therapy.

## Review

Methods

The protocol of this umbrella review was registered in the International Prospective Register of Systematic Reviews (PROSPERO) database (CRD42024564150) and developed according to the Preferred Reporting Items for Systematic Reviews and Meta-Analyses (PRISMA) guidelines.

Search Strategy

A comprehensive literature search was conducted in four databases - PubMed, Scopus, EBSCOhost, and Cochrane - to identify systematic reviews and meta-analyses published from 2016 up to July 2024. A manual search and a review of grey literature were also conducted. To find the pertinent systematic reviews, the search strategy (((guided endodontics) OR (static navigation)) OR (dynamic navigation)) AND (systematic review) was employed. The first step of the review process involved two independent reviewers, Alpa Gupta and Aakansha Puri, screening the titles and abstracts. The second stage entailed reviewing the complete text to identify the relevant reviews. Disagreements and uncertainties over the inclusion of the reviews were resolved by discussing them with the third reviewer, Dax Abraham.

Eligibility Criteria

The following systematic reviews and meta-analyses were included: studies that focused on guided endodontics; randomized controlled trials (RCTs), randomized clinical trials, case series, and case reports written in English. The following studies were excluded: reviews, non-English language articles, in vitro studies, ex vivo studies, randomized experimental (in vitro) studies, animal studies, narrative reviews, expert opinions, short communications, and editorials.

Data Extraction

Data extraction sheets included the first author's name, year of publication, title of the study, journal name, number of databases searched, country of the first author, search period, language, whether a meta-analysis was performed, number of studies included, study design, endodontic application, tooth involved, quality, outcome evaluation method, and instrument of quality assessment. The success of endodontic treatment was evaluated based on: follow-up for tooth structure loss using CBCT analysis; angle deviation of the bur tip in conventional and guided endodontics, as determined by CBCT; and clinical and radiological evaluation of the periapical lesion and its relationship to the pain scale.

Methodological Quality Assessment

A Measurement Tool to Assess Systematic Reviews 2 (AMSTAR 2) criteria - a 16-item checklist - was used as a measurement instrument to evaluate systematic reviews and meta-analyses and assess the quality of the included studies. Each study received a score based on whether the answer was "Yes," "No," or "Partial Yes." The systematic reviews were classified as high quality, moderate quality, low quality, and severely low quality based on the overall individual score of the research.

Results

Study Selection

**Figure 1:**
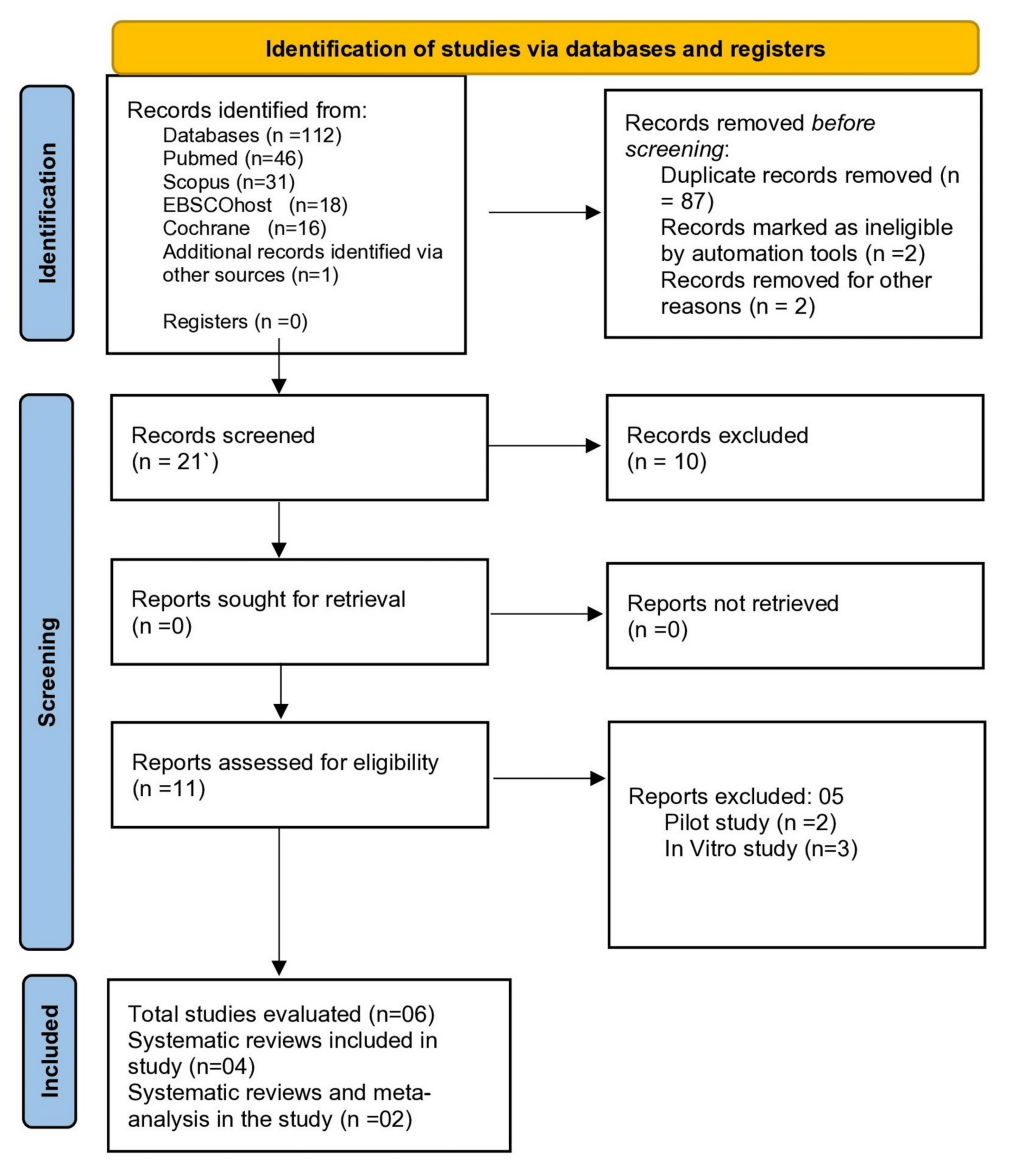
PRISMA flow diagram PRISMA: Preferred Reporting Items for Systematic Reviews and Meta-Analyses

The literature search identified a total of 112 studies in the initial phase (title/abstract) (Figure 1). Eighty-seven duplicates and two records marked as ineligible by automation tools were excluded. Two records were removed for other reasons. Further, 11 studies were assessed for eligibility criteria, of which five were excluded (pilot studies: two, in vitro studies: three). Finally, six systematic reviews were included, of which two studies had conducted meta-analyses.

Characteristics of the Included Reviews

Tables 1-2 display the characteristics of the six included studies. Four electronic databases - PubMed, Scopus, EBSCOhost, and Cochrane - along with grey literature, were searched from the inception of guided endodontics up to July 2024.

**Table 1:** Characteristics of the included studies

S. No	Author and year	Title	Name of the journal	Database searched	Country of the first author	Search period	Language	Meta-analysis performed	Number of included studies
1	Peña-Bengoa et al. (2023) [3]	Effectiveness of Guided Endodontics in Locating Calcified Root Canals: A Systematic Review	Clinical Oral Investigations	PubMed, Scopus, EBSCOhost	Chile	Upto July 2022	English	No	45 studies were included. Of them, 21 were clinical case reports, 11 case series, 12 ex vivo studies, and 1 cohort study
2	Zubizarreta-Macho et al. (January 2021) [4]	Efficacy of Computer-Aided Static Navigation Technique on the Accuracy of Endodontic Microsurgery: A Systematic Review and Meta-Analysis	Journal of Clinical Medicine	PubMed, Scopus, EBSCOhost	Spain	Up to April 2020	English	Yes, 7 articles were included meta-analysis	Of the 7 articles included, three were experimental trials, 3 were case series and 1 was a clinical trial
3	Zubizarreta-Macho et al. (March 2021) [5]	Effect of Computer-Aided Navigation Techniques on the Accuracy of Endodontic Access Cavities: A Systematic Review and Meta-Analysis	Biology (Basel)	PubMed, Scopus, EBSCOhost	Spain	Up to April 2020	English	Yes, 13 studies were included in the meta-analysis	Of the 13 articles included, 8 were experimental trials, 4 were case series, and 1 was a clinical trial
4	Moreno-Rabié et al. (2020) [6]	Clinical Applications, Accuracy and Limitations of Guided Endodontics: A Systematic Review	International Endodontic Journal	PubMed, Scopus, EBSCOhost	Leuven	Up to April 25, 2019	English	No	22 studies included, 15 case reports of which 11 of them corresponded to guided endodontic access cavity, 4 were related to guided endodontic surgery, and 1 observational study (50 patients)
5	Vasudevan et al. (2022) [7]	Dynamic Navigation in Guided Endodontics: A Systematic Review	Europian Endodontic Journal	PubMed, Scopus, EBSCOhost	India	Up to July 2021	English	No	14 studies were included of which 3 were case reports and 11 in-vitro studies
6	Poirazi et al. (2023) [8]	Post Removal Using Guided Endodontics: A Systematic Review of the Literature	European Journal of Dental and Oral Health	Open Grey literature	Greece	Up to July 2023	English	No	A total of 9 studies were included. Of those, 7 were case reports and two were in vitro studies

**Table 2:** Characteristics of the included studies

S. No	Author and year	Title	Study design - included studies	Endodontic application	Tooth involved	Outcome evaluation method	Instrument of quality assessment	Conclusion
1	Peña-Bengoa et al. (2023) [3]	Effectiveness of Guided Endodontics in Locating Calcified Root Canals: A Systematic Review	Observational studies, ex vivo studies, clinical case reports, and case series	Static endodontic guides were used for the location of calcified canals	The case reports and case series were analyzed together. Among the 32 studies, 21 used endodontic guidance in anterior teeth, of which 2 were in dens invaginatus and 1 in teeth with dentin dysplasia. The remaining 6 studies included the treatment of both maxillary and mandibular posterior teeth. In 3 studies, the use of endodontic guidance in anterior and posterior teeth was indistinct.	Measured based on drill diameter used, root canal localization, and complications associated	Joanna Briggs Institute critical appraisal tool	Guided endodontics is an effective and precise technique to access the permeable portion of calcified canals.
2	Zubizarreta-Macho et al. (January 2021) [4]	Efficacy of Computer-Aided Static Navigation Technique on the Accuracy of Endodontic Microsurgery: A Systematic Review and Meta-Analysis	In vitro randomized experimental trials, randomized clinical trials, clinical trials, and case series	Apex location rate of the computer-aided static navigation techniques applied to endodontic microsurgery	Maxillary anterior teeth, mandibular molars	Clinical and radiographic assessment	Jadad scale for methodological quality assessment of clinical trials	The root apex location success rate stated at 96.8% (confidence interval (CI): 93.0-100%) of the cases performed through a computer-aided static navigation technique. The study showed a root apex location success rate 27 times higher than conventional endodontic microsurgery procedures.
3	Zubizarreta-Macho et al. (March 2021) [5]	Effect of Computer-Aided Navigation Techniques on the Accuracy of Endodontic Access Cavities: A Systematic Review and Meta-Analysis	Randomized experimental trials, clinical trials, and case series	Endodontic access cavities were performed using computer-aided techniques (static or dynamic navigation techniques)	Partially or completely calcified anterior teeth, maxillary posterior teeth	Precise root canal location and clinical and radiographic assessment	Jadad scale for methodological quality assessment of clinical trials	The root canal location success rate started at 98.1% (CI: 95.7-100%) of the cases performed through a computer-aided navigation technique. No statistically significant differences were found between computer-aided static navigation techniques (success rate: 98.5%) and computer-aided dynamic navigation techniques (success rate: 94.5%) (Q test = 0.57; p = 0.451), nor between in vitro studies (success rate: 96.2%) and in vivo studies (success rate: 100%).
4	Moreno-Rabié et al. (2020) [6]	Clinical Applications, Accuracy and Limitations of Guided Endodontics: A Systematic Review	Case reports, in vitro and ex vivo studies, observational study	Guided endodontics access cavity preparation, canal localization in pulp canal obliteration in need of the post and guided surgery	9 articles performed access cavities in anterior single-rooted teeth (7 of them were treatments for calcified canals, 2 on teeth with anomalies such as dens invaginatus and dens evaginatus). The rest of the access cavities were made in calcified canals of maxillary and mandibular molars. Periapical surgery was performed on incisors, canines, premolars, and molars.	Precise apex localization, complication, drill path precision, clinical and radiograph assessment	CARE guideline (Case Report guideline)	All articles describe guided access cavity preparation and guided surgery as being highly accurate and successful techniques when comparing the drilled path to the planned treatment.
5	Vasudevan et al. (2022) [7]	Dynamic Navigation in Guided Endodontics: A Systematic Review	In-vitro or ex-vivo studies that assessed the accuracy of treatment, and case reports that assessed efficiency, accuracy, and limitations of diagnostic navigation system (DNS)	Access cavity preparation, pulp canal obliteration, endodontic retreatment, and microsurgery using dynamic navigation	Teeth with difficult access to root canals (calcified canals/teeth with malformations) or teeth requiring endodontic microsurgery or other clinical scenarios.	Successful localization, time taken, iatrogenic error, clinical and radiographic assessment	Joanna Briggs Institute (JBI) critical appraisal checklists	Challenging clinical situations like pulp canal obliteration, conservative access preparation, endodontic retreatment, and microsurgery can be managed efficiently with fewer iatrogenic errors in a shorter time using DNS.
6	Poirazi et al. (2023) [8]	Post Removal Using Guided Endodontics: A Systematic Review of the Literature	Case reports	Guided endodontics for guides for dental post-removal	Human teeth with dental post. Teeth included were: maxillary incisors, maxillary bicuspids, maxillary molars, mandibular bicuspids, and mandibular molars.	Ability to remove the dental post without causing severe complications such as a fracture or a perforation of the root	CARE guidelines (Case Report guideline)	Guided endodontics was shown to be an effective technique for the removal of dental posts with a low risk of iatrogenic errors. In all case reports the post was successfully removed with no complications (100% success rate).

Table 3 shows a summary of the studies in which a meta-analysis was conducted.

**Table 3:** Summary of the meta-analysis

S. No.	Author and year	Number of samples	Meta-analysis	Estimate 95%	Total studies included	I^2^%	Outcome assessed
1	Zubizarreta-Macho et al. (January 2021) [4]	126	Yes	Yes: 0.97 (0.93; 1)	7	2.4%	Apex location prediction ranged from 91.4% to 100%
		Experimental = 105; total = 95	Yes	Yes: 27.67 (11.25; 68.07)	3	0%	Success rate of the root canal location comparing the computer-aided navigation technique and control group
		Experimental = 81; control = 71	Yes	Yes: -1.39 (-1.71; -1.07)	2	0%	Apex measurement error using computer-aided navigation with respect to the control group
2	Zubizarreta-Macho et al. (March 2021) [5]	307	Yes	Yes: 0.98 (0.96; 1)	13	25%	Success rate of root canal location ranged from 93.3% to 100%
		-	Yes	Yes: Static navigation: 0.98 (0.96; 1); Dynamic navigation: 0.94 (0.97; 1)	14	-	Root canal success rate of computer-aided navigation technique. Static navigation showed a success rate of 98.5% and dynamic navigation 94.5%
		-	Yes	Yes: In-vitro: 0.96 (0.92; 1); In-vivo: 1.00 (0.97; 1)	14	0%	Success rate between study types (in-vitro, in-vivo)
		Experimental = 44; control = 44	Yes	Yes: 13.7 (3.48; 49.13)	3	0%	Comparison between computer-aided navigation and control group

Methodological Quality of Assessment

AMSTAR 2 was used for the quality assessment of the studies. Scores were assigned according to the checklist items (Table 4).

**Table 4:** AMSTAR 2 score for the included studies AMSTAR 2: A Measurement Tool to Assess Systematic Reviews 2

S. No.	Questions	Peña-Bengoa et al. (2023) [3]	Zubizarreta-Macho et al. (January 2021) [4]	Zubizarreta-Macho et al. (March 2021) [5]	Moreno-Rabié et al. (2020) [6]	Vasudevan et al. (2022) [7]	Poirazi et al. (2023) [8]
1	Did the research questions and inclusion criteria for the review include the components of PICO?	Partial yes	Yes	Yes	Yes	Yes	Yes
2	Did the report of the review contain an explicit statement that the review methods were established prior to the conduct of the review and did the report justify any significant deviations from the protocol?	Partial yes	Partial yes	Yes	Yes	Partial yes	Yes
3	Did the review authors explain their selection of the study designs for inclusion in the review?	Yes	Yes	Yes	Yes	Yes	No
4	Did the review authors use a comprehensive literature search strategy?	Yes	Yes	Partial yes	Yes	Yes	Yes
5	Did the review authors perform study selection in duplicate?	Yes	Yes	Yes	Yes	Yes	Yes
6	Did the review authors perform data extraction in duplicate?	Yes	Yes	Yes	Yes	Yes	Yes
7	Did the review authors provide a list of excluded studies and justify the exclusions?	Partial yes	Partial yes	Partial yes	Partial yes	Yes	Yes
8	Did the review authors describe the included studies in adequate detail?	Yes	Partial yes	Partial yes	Yes	Yes	Partial yes
9	Did the review authors use a satisfactory technique for assessing the risk of bias (RoB) in individual studies that were included in the review?	Yes	Partial yes	Yes	Yes	Yes	Partial yes
10	Did the review authors report on the sources of funding for the studies included in the review?	No	Yes	Yes	No	Yes	No
11	If meta-analysis was performed did the review authors use appropriate methods for statistical combination of results?	N/A	Yes	Yes	N/A	N/A	N/A
12	If meta-analysis was performed, did the review authors assess the potential impact of RoB in individual studies on the results of the meta-analysis or other evidence synthesis?	N/A	Yes	Yes	N/A	N/A	N/A
13	Did the review authors account for RoB in individual studies when interpreting/ discussing the results of the review?	Yes	Yes	Yes	Yes	Yes	Yes
14.	Did the review authors provide a satisfactory explanation for, and discussion of, any heterogeneity observed in the results of the review?	Yes	Yes	Yes	Yes	Yes	Yes
15	If they performed quantitative synthesis did the review authors carry out an adequate investigation of publication bias (small study bias) and discuss its likely impact on the results of the review?	N/A	Yes	Yes	N/A	N/A	N/A
16	Did the review authors report any potential sources of conflict of interest, including any funding they received for conducting the review?	Yes	Yes	Yes	Yes	Yes	Yes

Peña-Bengoa et al. (2023) reported a high success rate in locating calcified canals. Only two articles showed failures in guided endodontics. The evidence from the pooled studies suggested that guided endodontics is an accurate and safe procedure for locating root canals. However, an average drill tip deviation of 0.46 mm was noted, which may lead to undesirable loss of healthy dentinal structure. This deviation is not influenced by the operator but by factors such as the guide design, making guided endodontics less suitable for teeth with complex anatomy. The study scored moderate quality (10.5/16) according to the AMSTAR 2 criteria, due to an inadequate description of the heterogeneity of data, and because the funding sources were not recorded. Nine case reports were categorized as having a moderate risk of bias, and 12 studies had a low risk of bias. Upon evaluation of the 11 case series, all displayed a low risk of bias; however, only one study showed a medium risk of bias. Most studies lacked adequate follow-up, thus limiting the assessment of long-term success. Potential publication bias needed to be considered [3].

The systematic review and meta-analysis conducted by Zubizarreta-Macho et al. (January 2021) received a score of 14/16, which was considered high quality. The studies were combined and assessed using the random effects model with the inverse variance method. The success rate for apex location using computer-aided SN was 96.8%, with a confidence interval ranging from 93% to 100%. The asymmetry of the funnel plot was addressed using the trim and fill method. When compared to conventional endodontic microsurgery, the success rate was 27 times greater [4].

In another study conducted by Zubizarreta-Macho et al. (March 2021), an AMSTAR 2 score of 14.5/16 (high quality) was noted. Using an inverse variance technique and the random effects model, 13 studies were integrated. The success percentage for root canal location was determined to be 98.1%. For these studies, no heterogeneity was found [5].

Moreno-Rabié et al. (2020) scored an AMSTAR 2 score of 11.5/16 (moderate quality). Of the 15 case reports included, four were related to guided endodontic surgery, and 11 focused on access cavities using guided endodontics. Nine case reports involved anterior single-rooted teeth, primarily treating calcified canals, while the remaining included molars/teeth with anomalies. An observational study including 50 patients demonstrated acceptable precision in all endodontic procedures. According to the quality assessment, adherence varied among studies, with pre-clinical studies showing the lowest adherence and case reports showing the highest adherence. The quality assessment showed variable compliance across studies, with the highest adherence in case reports and lower adherence in pre-clinical studies. The sources of funding were not specified. Although the study addressed the possibility of bias, it did not apply to all the investigations [6].

Vasudevan et al. (2022) showed an AMSTAR 2 score of 12.5/16 (high quality). Three case reports showed a low risk of bias, with a mean score of 83.34%. Two studies, with an average score of 84.09%, indicated a moderate risk of bias, whereas nine in vitro studies had a low risk of bias. Meta-analysis was not applied to this study due to the heterogeneity of data among the included articles, including sample size, type of teeth, outcome measures, and other methodological differences [7].

A study by Poirazi et al. (2023) was judged to be of moderate quality (10/16). Heterogeneity and low evidence were acknowledged; however, the follow-up times for the included studies were not described. The prognosis, patient perspective, intervention adherence and tolerability, and consent were not met by the included case studies. Six case reports and two in vitro experiments, both categorized as low-level evidence, are included in this review [8].

Discussion

The evolution of endodontic techniques requires standardized evidence synthesis and is critical for directing current clinical practice. Guided endodontics uses advanced navigation technologies to improve the precision of endodontic procedures and has a major impact on contemporary dentistry. This approach has the potential to enhance root canal therapy success, including the precision with which calcified root canals are located, tooth structure preservation, and overall procedure success. However, the data on the efficacy and clinical benefits of guided endodontics vary across several studies, with different methodologies and outcomes.

The increased focus on guided endodontics can be attributed to assertions that it has a significant impact on endodontic treatment results [3]. Numerous studies list the advantages of guided endodontics, such as improved precision, less tooth structure loss, and better treatment outcomes with a favorable long-term prognosis [9]. Nevertheless, contradictory data have been found in the literature, with some research demonstrating a large improvement and others demonstrating little or no change when compared to conventional procedures [10]. Therefore, the goal of this umbrella review was to bring together systematic reviews and meta-analyses in order to present a thorough understanding of the effectiveness of guided endodontics in enhancing the results of endodontic treatment and directing future clinical practice.

Outcomes Assessed in the Systematic Reviews

Six systematic reviews were included, addressing the following outcomes: Peña-Bengoa et al. (2023) [3] - the success rate of guided endodontics in accurately locating calcified root canals; Zubizarreta-Macho et al. (January 2021) [4] - the accuracy of SN techniques in endodontic microsurgery; Zubizarreta-Macho et al. (March 2021) [5] - the accuracy of computer-aided navigation techniques in endodontic access cavities; Moreno-Rabié et al. (2020) [6] - clinical success rates, accuracy in locating and treating canals, and limitations of guided endodontics; Vasudevan et al. (2022) [7] - the effectiveness of DN systems in endodontic treatments; Poirazi et al. (2023) [8] - the efficacy and success rate of guided endodontics in post-removal procedures.

Peña-Bengoa et al. (2023) [3] evaluated the efficiency of guided endodontics to locate calcified root canals. Forty-five studies were included, mainly observational and ex vivo studies, along with 21 case reports and 11 case series. The majority of the studies reported high success rates, with only two failures. The Joanna Briggs Institute critical appraisal tool was applied to assess the quality of evidence for case reports [11,12]. Case reports and case series were analyzed together; 21 out of the 32 studies included treatment with guided endodontics in anterior teeth, two of which included dens invaginatus and one tooth with dentin dysplasia. The remaining six studies treated maxillary and mandibular posterior teeth. Loureiro et al. (2020) found that guided endodontics significantly preserved the volume of dental tissue in maxillary molars compared to conventional methods. Small drill diameters were found to be more successful at preserving tissues, while ultrasonic tips with endodontic guides provided an excellent option for small teeth that required critical preservation of tooth structure [13,14]. Studies showed that using a small voxel size (0.075-0.8 mm) in CBCT allows for clear visualization of the root canal, as it provides better resolution, making them ideal for posterior teeth with high density and many root canals.

Numerous studies also analyzed the planning time for treating cases with guided endodontics. The time taken varied based on multiple factors, including printing type, management of software, and printer used [12,15]. Connert et al. (2017) found that the average planning time was 9.4 minutes, with a range of 7-12.8 minutes [16]. All authors agreed that using guided endodontics to treat calcified root canals minimizes the risk of accidents, tissue loss, and working time. However, the quality of evidence is limited due to a lack of patient follow-up and population description, impacting future prevalence studies. Despite the low risk of bias, case reports and series have potential publication bias, emphasizing the need for high-quality studies.

Zubizarreta-Macho et al. (January 2021) conducted a systematic review and meta-analysis to evaluate the precision with which computer-aided navigation can locate the root apex in the course of endodontic microsurgery [4]. Real-time drilling guidance is now possible due to the development of computer-aided DN techniques employing optical triangulation tracking systems, as reported by Mediavilla Guzmán et al. [17]. Zubizarreta-Macho et al. (January 2021) included seven studies and found that the success rate for root apex location using computer-aided SN was 96.8% (confidence interval (CI): 93.0-100%), with a forecasted range of 91.4% to 100%. Heterogeneity was not seen in any of the investigations (Q-test = 6.15; p = 0.407; I² = 2.4%). The methodology was 27 times more effective than traditional procedures (Q-test = 0.80; p = 0.671; I² = 0%), with a statistically significant odds success ratio of 27.7 (95% CI: 11.3-68.1; z-test = 7.23; p < 0.0001).

Zubizarreta-Macho et al. (March 2021) [5] examined how computer-aided navigation methods affected endodontic access cavity accuracy. The study focused on endodontic access cavities treated with SN or DN systems and comprised randomized experimental trials, clinical trials, and case series involving two or more patients. Although both navigation techniques employ CBCT datasets, SN requires computer-aided design and manufacturing of surgical templates through rapid prototyping. On the other hand, computer-aided DN methods require an optical triangulation tracking system that employs stereoscopic motion-tracking cameras to guide the drilling process in real time at the intended angle, pathway, and depth of the endodontic access cavities. With sample sizes ranging from 2 to 60, the included studies consisted of eight experimental experiments, four case series, and one clinical trial. Methodological quality was assessed using the Checklist for Reporting In-vitro Studies (CRIS) scale for in vitro studies and the Jadad scale for clinical studies, revealing limitations in randomization and blinding. The only clinical trial included scored 0 on the Jadad scale, indicating poor quality. The meta-analysis used a random effects model, analyzing root canal location success rates and odds ratios with a 95% CI. The overall root canal location success rate was 98.1% (CI: 95.7-100%), with no significant heterogeneity between studies (Q-test = 17.3; p = 0.185; I² = 25%). No significant differences were found between static (98.5% success) and dynamic (94.5% success) navigation techniques. In vitro studies had a success rate of 96.2% (CI: 92.4-100%), while in vivo studies achieved 100% (CI: 97.3-100%). It was concluded that computer-aided navigation techniques significantly enhance the accuracy of endodontic access cavities, demonstrating high success rates and effective root canal location. However, it was also noted that, although SN has been used for performing endodontic microsurgeries, in clinical situations where there are limited mouth openings or posterior teeth with restricted access, SN might be impractical. These challenges can be resolved by DN, as real-time adjustments and direct visualization of the access cavity can be done, overcoming any potential design or manufacturing errors of SN templates.

Moreno-Rabié et al. (2020) [6] conducted a systematic review aimed at assessing the clinical applications, accuracy, and limitations of guided endodontic treatment. A total of 22 articles were included, comprising 15 case reports, six pre-clinical studies (in vitro and ex vivo), and one observational study. Of the 15 case reports, 11 focused on guided endodontic access cavities and four on guided endodontic surgery. Access cavities were primarily in anterior, single-rooted teeth. Most of the cases treated calcified canals, while some involved conditions such as dens invaginatus and dens evaginatum. The remaining cases included treatment of calcified canals in maxillary and mandibular molars. Guided endodontic surgery was performed in four case reports. The periapical operations were carried out on different types of teeth. Additionally, in an observational study of 50 patients treated using this method, Buchgreitz et al. (2019) found that a reasonable deviation of the bur can be considered "acceptable" precision. "Acceptable" was used when there was some deviation, but the canal could still be located and instrumented, and the apical lesion healed upon follow-up [18].

Vasudevan et al. (2022) [7] evaluated the applications, accuracy, benefits, and limitations of DN systems in endodontics. Three case reports and 11 in vitro studies were selected for this review. Quality assessments revealed a low risk of bias, with an average score of 83.34% for case reports and 84.09% for in vitro studies. DN systems were utilized for various applications, including access cavity preparation, pulp canal obliteration, endodontic retreatment, and microsurgery. A meta-analysis was not possible due to heterogeneity. Accuracy was evaluated by comparing two-dimensional (2D) and three-dimensional (3D) deviations between planned and actual drill paths, with mean 3D lateral deviations of 0.67 mm coronally and 0.9 mm apically, and angular deviations of 2.50° [19]. The mean 2D horizontal deviation for endodontic access was found to be 0.9 mm, while the angular deviation was 1.7° [20]. DN systems significantly reduced iatrogenic errors, which include misaligned access, gouging, unsuccessful canal location, perforation, and incomplete root-end resection. Treatment times ranged from 11.5 seconds for ultraconservative access preparation to under 45 minutes for endodontic microsurgery [21,22]. Chairside time was significantly reduced compared to freehand techniques. The study concluded that challenging clinical cases can be effectively and safely managed with fewer iatrogenic errors using DN systems, as they allow real-time feedback and retracing during treatment. This is achieved through multiple motion-tracking devices and cameras attached to the dental handpiece as well as the patient.

Poirazi et al. (2023) [8] demonstrated a high success rate for guided endodontics in the removal of fiber posts. To quantify the effectiveness, the percentage of cases with successful removal of the posts, without severe complications like fractures or root perforations, was calculated. The success rate in the case reports reviewed was 100%, meaning all cases of post-removal were successful and uneventful. This result suggests that guided endodontics is an excellent tool for removing fiber posts within a clinical setting [23-28].

Quality of Evidence and Methodological Considerations

The AMSTAR 2 assessment of the included studies revealed variability in methodological rigor and quality. The Peña-Bengoa et al. (2023) [3] study received a moderate score, indicating moderate quality. The main areas of concern included the lack of clarity in reporting conflicts of interest and insufficient detail in the study selection process. Similarly, the study by Moreno-Rabié et al. (2020) [6], which scored 11.5/16, exhibited methodological limitations, particularly in the areas of risk of bias assessment and transparency in study selection criteria. These limitations underline the need for more rigorous study designs and transparent reporting to enhance the reliability of systematic reviews in this field.

In contrast, the studies by Zubizarreta-Macho et al. (2021) [4,5] received higher AMSTAR 2 scores (14/16 and 14.5/16, respectively), reflecting their strong methodological rigor, comprehensive risk of bias assessments, and thorough reporting of study selection and data extraction processes. These high-quality reviews provide a more robust evidence base, supporting the efficacy of guided endodontic techniques.

Limitations and future directions

While guided endodontics shows promise in enhancing the precision and success of endodontic procedures, the current body of evidence is not without its limitations. The variability in study quality, as indicated by the AMSTAR 2 assessments, suggests that the conclusions drawn from some of these reviews should be interpreted with caution. Additionally, the reliance on advanced imaging technologies and high-tech equipment may limit the accessibility and generalizability of guided endodontic techniques in routine clinical practice.

Further research, particularly high-quality RCTs and long-term clinical studies, is needed to validate the findings of these systematic reviews and to establish standardized protocols for the implementation of guided endodontics. Future studies should also focus on cost-effectiveness analyses and the development of user-friendly guided systems that can be more widely adopted in clinical settings. Despite the promising potential of guided endodontics in enhancing procedural accuracy and outcomes, the current evidence presents certain limitations. Studies showed that the average drill tip deviation was 0.46 mm, potentially causing unwanted wear of healthy tissue. Zenhder et al. (2016) [29] and Connert et al. (2017) [16] also reported deviations, highlighting the reproducibility of guided endodontics. Nayak et al. (2018) [30] pointed out that guide design could cause deviations, making the technique less suitable for complex anatomies like C-shaped canals.

The variability in methodological quality across the included systematic reviews, as reflected by their AMSTAR 2 scores, suggests that the evidence supporting guided endodontics is not uniformly robust. For instance, studies such as those by Peña-Bengoa et al. (2023) [3] and Poirazi et al. (2023) [8], which received moderate AMSTAR 2 scores, highlight gaps in methodological rigor, particularly in areas such as bias assessment and study selection transparency. These limitations underscore the necessity for more stringent and standardized methodological approaches in future systematic reviews and meta-analyses. Furthermore, the reliance on advanced imaging and navigation technologies, while beneficial for accuracy, introduces practical challenges, including high costs, a steep learning curve, and limited accessibility in routine clinical practice. The integration of these technologies into standard care requires not only validation through high-quality RCTs but also consideration of cost-effectiveness and ease of use. Future research should focus on optimizing these technologies to make them more accessible and user-friendly, thereby facilitating their broader adoption in clinical settings.

## Conclusions

Guided endodontics facilitates the accuracy and predictability of the endodontic procedure. The results of this umbrella review demonstrate that guided endodontics has notable advantages over conventional techniques, including improved precision, reduced procedural errors, and better clinical outcomes in locating calcified root canals and in carrying out endodontic microsurgical procedures. However, certain limitations were observed, concerning the varying quality of evidence among the studies, drill tip variations, and particularly case reports as well as case series. Guided endodontics has shown some promise in the management of complex endodontic cases, but further and higher-quality research is needed to ascertain its full long-term effectiveness and to establish its place in everyday clinical practice.
